# Patient Online Record Access in English Primary Care: Qualitative Survey Study of General Practitioners’ Views

**DOI:** 10.2196/43496

**Published:** 2023-02-22

**Authors:** Charlotte Blease, John Torous, Zhiyong Dong, Gail Davidge, Catherine DesRoches, Anna Kharko, Andrew Turner, Ray Jones, Maria Hägglund, Brian McMillan

**Affiliations:** 1 Division of General Medicine Beth Israel Deaconess Medical Center Harvard Medical School Boston, MA United States; 2 Digital Psychiatry, Department of Psychiatry Beth Israel Deaconess Medical Center Harvard Medical School Boston, MA United States; 3 Centre for Primary Care and Health Services Research University of Manchester Manchester United Kingdom; 4 Healthcare Sciences and e-Health Department of Women's and Children's Health Uppsala University Uppsala Sweden; 5 School of Psychology Faculty of Health University of Plymouth Plymouth United Kingdom; 6 Centre for Academic Primary Care Population Health Sciences University of Bristol Bristol United Kingdom; 7 National Institute for Health Research Applied Research Collaboration West University Hospitals Bristol and Weston NHS Foundation Trust Bristol United Kingdom; 8 School of Nursing and Midwifery Faculty of Health University of Plymouth Plymouth United Kingdom

**Keywords:** electronic health records, attitudes, general practice, patients, online record access, open notes, opinions, primary care, qualitative research

## Abstract

**Background:**

In 2022, NHS England announced plans to ensure that all adult primary care patients in England would have full online access to new data added to their general practitioner (GP) record. However, this plan has not yet been fully implemented. Since April 2020, the GP contract in England has already committed to offering patients full online record access on a prospective basis and on request. However, there has been limited research into UK GPs’ experiences and opinions about this practice innovation.

**Objective:**

This study aimed to explore the experiences and opinions of GPs in England about patients’ access to their full web-based health record, including clinicians’ free-text summaries of the consultation (so-called “open notes”).

**Methods:**

In March 2022, using a convenience sample, we administered a web-based mixed methods survey of 400 GPs in the United Kingdom to explore their experiences and opinions about the impact on patients and GPs’ practices to offer patients full online access to their health records. Participants were recruited using the clinician marketing service Doctors.net.uk from registered GPs currently working in England. We conducted a qualitative descriptive analysis of written responses (“comments”) to 4 open-ended questions embedded in a web-based questionnaire.

**Results:**

Of 400 GPs, 224 (56%) left comments that were classified into 4 major themes: increased strain on GP practices, the potential to harm patients, changes to documentation, and legal concerns. GPs believed that patient access would lead to extra work for them, reduced efficiency, and increased burnout. The participants also believed that access would increase patient anxiety and incur risks to patient safety. Experienced and perceived documentation changes included reduced candor and changes to record functionality. Anticipated legal concerns encompassed fears about increased litigation risks and lack of legal guidance to GPs about how to manage documentation that would be read by patients and potential third parties.

**Conclusions:**

This study provides timely information on the views of GPs in England regarding patient access to their web-based health records. Overwhelmingly, GPs were skeptical about the benefits of access both for patients and to their practices. These views are similar to those expressed by clinicians in other countries, including Nordic countries and the United States before patient access. The survey was limited by the convenience sample, and it is not possible to infer that our sample was representative of the opinions of GPs in England. More extensive, qualitative research is required to understand the perspectives of patients in England after experiencing access to their web-based records. Finally, further research is needed to explore objective measures of the impact of patient access to their records on health outcomes, clinician workload, and changes to documentation.

## Introduction

In 2022, NHS England announced plans to ensure that all adult primary care patients in England would have full online access to new data added to their general practitioner (GP) record [1]. This decision has since been paused [2]. The original aim of the announcement was to permit patients aged ≥16 years to be able to read all new information added to their primary care electronic health record via web-based services such as the NHS (National Health Service) app [3] with access encompassing test and laboratory results; lists of medications; coded records about problems, diagnoses, and treatments; and the free-text entries written by clinical staff about patients’ consultations. From April 2020, the GP contract in England has committed primary care practices to offer patients full online access on a prospective basis and on request [4]. However, the figures suggest that uptake by practice has been slow, and many patients were unaware that they could request access [5,6]. For example, in March 2022, only 13% were able to view detailed coded records [7]. Under the new NHS England announcement, access will be automatic without requiring patients to submit a request to their GP surgery.

Similar policies enabling patient online record access (ORA) have already been rolled out in other countries, including in Scandinavia and the United States [8]. For example, since 2005 in Estonia and between 2012 and 2018 in Sweden, patients were given online access to their electronic health records [9]. Starting April 2021, new federal rules mandated, with few permitted exemptions, all patients in the United States be offered rapid access to their full electronic record without charge [10,11]. Studies have shown that most patients welcome access and derive many benefits, including enhanced understanding and recall of their care plan, trusting their physician more, and doing a better job taking their medications [5,12-22]. However, clinicians, especially those with limited experience in practice, tend to be cautious or resistant, believing that access will create more work, and patients will be confused and worried about what they read [23-31].

Although there is now substantial survey research into clinicians’ views of ORA in Scandinavia and the United States [12,23-31], whether GPs in England view ORA similarly to their counterparts in other countries remains unknown. Systemic differences in health organizations suggest caution about drawing generalizations across national boundaries. In the last decade, for example, the United Kingdom had a lower level of capital investment in health care compared with the other 14 European Union countries [32]. The United Kingdom also has one of the lowest numbers of doctors per capita in Europe [33]. Compared with the United States, patients in the United Kingdom and Europe are also more likely to have a longstanding relationship with their primary care physician and are more likely to see primary care doctors out of office hours [34]. It remains to be seen whether these factors foster greater workplace pressure, including perceived additional stressors, on British doctors, which might render GPs in England more skeptical about ORA than clinicians in other countries.

Only a few recent studies in England have explored patients’ and clinicians’ views about “ORA,” including to the free-text entries written by doctors about patients’ consultations (“open notes”) [35,36]. For example, in 2022, Turner et al [6] conducted a qualitative study of 16 general practice staff with experience of ORA and reported multiple concerns, including the effects on documentation quality, patient safety, and practice workloads. In a study by Louch et al [37] of 19 primary care staff involved in a variety of clinical and nonclinical roles, respondents generally appeared in favor of ORA but were uncertain about the impact on patient-clinician relationships and reported concerns about patient and clinician safeguarding. Research in England on clinicians’ experiences with ORA is limited to in-depth, small-scale, qualitative studies. Both Louch et al [37] and Turner et al [6] conducted their studies in 2019 at a time when experiences with ORA were limited, as it was not the default position for practices. Therefore, we aimed to address this gap by sampling a larger number of registered GPs working in England to investigate their experiences and opinions regarding the impact of ORA on patient care and their practice.

## Methods

### Main Survey

We conducted an anonymous nationwide web-based survey of GPs in England (n=400; Multimedia Appendix 1). We used a convenience sample to solicit the opinions of participants using the membership of the clinician marketing service Doctors.net.uk [38]. This is the largest web-based medical network in the United Kingdom, with 248,326 (69.9%) registered doctors out of a total of 355,250 British doctors. Approximately, 21,250 (57.82%) GPs out of a total of 36,752 registered and working in the United Kingdom are active in the community during any 90-day period. Among those registered with Doctors.net.uk, a variable percentage of GPs active within the community also consented to being sent survey invitations via email. Depending on how GPs consented to receive survey invitations, our study was advertised via email or displayed on the Doctors.net.uk home pages of a quota sample of GPs between March 10 and 31, 2022. The sample was stratified according to sex, age, and geographic location using demographic information about registered GPs in England provided by the General Medical Council (GMC) in March 2022 [39]. Doctors.net.uk invited 720 GPs by email and also by invitations embedded in their Doctors.net.uk home pages; a further 2072 GPs were invited to participate only via links on their home pages. We obtained samples from Doctors.net.uk in previous studies using similar methods [40,41].

The study team adapted a mixed methods survey instrument originally developed to explore US primary care physicians’ views and experiences with open notes [24]. This survey was adapted in consultation with GPs in England and piloted with GP colleagues in the United Kingdom (n=5) to ensure face validity. The survey was timed to take approximately 5 minutes to complete.

### Qualitative Component

The survey instrument included 4 optional open-ended questions that allowed participants to respond in detail to the topic of the questionnaire (Textbox 1).

**Open comment questions.**

- Question 1: Please add any additional comments about the information that patients can access.
- Question 2: Do you have any additional comments about the impact on patients accessing their full GP health records online?
- Question 3: Do you have any additional comments on the impact of patient access to their full online health records on your practice?
- Question 4: Do you have any additional comments, anecdotes, and/or expectations to share about patients’ online access to their full online health records, including clinicians’ free-text entries?

Open-ended questions were strategically placed within the survey to minimize the risks of prompts excessively influencing participants’ responses in a negative or positive direction. Question 1 was embedded in the survey after a series of closed-ended questions about where GPs practiced and the percentage of patients in their practices that GPs estimated could access full online access to their health records (Multimedia Appendix 1). Question 2 was embedded after a series of 10 Likert-scale questions encompassing a range of both positively and negatively valenced items with respect to the effects of ORA on patient care, for example, “Among my patients who read their full GP health record on the web a majority will: (a) Better understand their health and medical conditions; (b) Worry more...” Question 3 was inserted after a series of 8 Likert-scale questions, which encompassed a balance of positive and negatively valenced items on the effects or ORA on GPs’ practice, for example, “I will be/already am less candid in my documentation” and “Medical care will be/is delivered more efficiently.” Finally, question 4 was embedded at the end of the survey after the participants’ demographic questions. Thematic qualitative data analysis was used to investigate these responses [42,43].

We carried out inductive thematic coding of the data [44]. Responses were analyzed by 2 members of the research team (CB and JT). CB is a philosopher of medicine and health care ethicist from the United Kingdom, and JT is an informatician and psychiatrist from the United States with experience in sharing online access to patients’ health records [45,46]. The comment transcripts were initially read numerous times to achieve familiarization with the participant responses. Next, an inductive coding process was used, in which brief descriptive labels (“codes”) were applied to each comment. Multiple codes were applied to the comments with multiple meanings. Comments and codes were reviewed and compared to investigate similarities and differences. First-order codes were grouped into second-order themes, which were further divided into third-order categories to provide a descriptive summary of responses. CB and JT met to discuss coding decisions, and subsequently, minor revisions were made.

### Ethics Approval and Informed Consent

Ethics approval for this survey was obtained from Beth Israel Deaconess Medical Centre, Harvard Medical School (protocol #2021P000626). Owing to the anonymity of this human subject study, the survey was deemed exempt from full ethical review. The invited participants were informed that no personally identifying data would be gathered and that they would be fully anonymous to the research team. All respondents provided informed consent before participating. On completion, respondents were recompensed for their time with £7.50 (US $8.80) worth of “1000 eSR” points via Doctors.net.uk, which are exchangeable for web-based shopping vouchers.

## Results

### Overview

Of the 720 GPs who received email and Doctors.net.uk home page invitations, 601 opened the email invite, and 102 clicked on the survey link, with 63 completing the survey (response rate: 63/720, 8.8%); the remainder (337/2072, 16.26%) accessed and completed the survey via their home page. Of the 400 GPs who responded, more (227/400, 56.8%) were male, and 84.8% (339/400) were aged ≥40 years (Table 1). The respondents were from all regions in England. Most (230/400, 57.5%) of our respondents worked between 21 and 40 hours per week. Our participants differed somewhat from the GPs in England registered with the GMC in March 2022. At the time, most (32,171/59,001, 54.53%) GPs in the registry were female. Our respondents were also older than those in the registry, where 75.2% (44,366/59,001) of GPs in England were aged ≥40 years. Proportional regional representation, however, in our sample was reasonably similar to the GMC registry with slight underrepresentation in the South West (8091/59001, 13.71% in the registry vs 45/400, 11.23% in our sample) and slight overrepresentation in the East of England (5552/59001, 9.41% vs 40/400, 10%) and the Midlands (10274/59001, 17.41% vs 77/400, 19.3%). The GMC does not collect the number of hours GPs work per week; therefore, it was not possible to compare participants using this metric.

**Table 1:** Characteristics of the respondents and their practices (N=400).

Characteristics			Values
**Sex, n (%)**			
	Female	161 (40.2)	
	Male	227 (56.8)	
	Prefer not to disclose	12 (3)	
**Age (years), n (%)**			
	30-39	61 (15.2)	
	40-49	196 (49)	
	50-59	99 (24.8)	
	≥60	44 (11)	
**Hours worked per week, mean (SD)**			
	0-20	65 (16.2)	
	21-40	230 (57.5)	
	≥41	105 (26.2)	
**Location of practice, n (%)**			
	London	64 (16)	
	South West	45 (11.2)	
	South East	64 (16)	
	West Midlands	42 (10.5)	
	East Midlands	35 (8.8)	
	East of England	40 (10)	
	Yorkshire and Humber	42 (10.5)	
	North East	16 (4)	
	North West	52 (13)	

A total of 224 (56% out of the 400 respondents) GPs left comments in response to at least 1 question (10,629 words in total). The comments were brief (1 phrase or 1-3 sentences). GP respondents who submitted comments to each question were not significantly different from those respondents who did not submit comments in terms of sex, age, whether they worked ≥40 hours per week, or whether they shared access to most of their patients (see Multimedia Appendix 2 for comparison).

**Figure 1:**
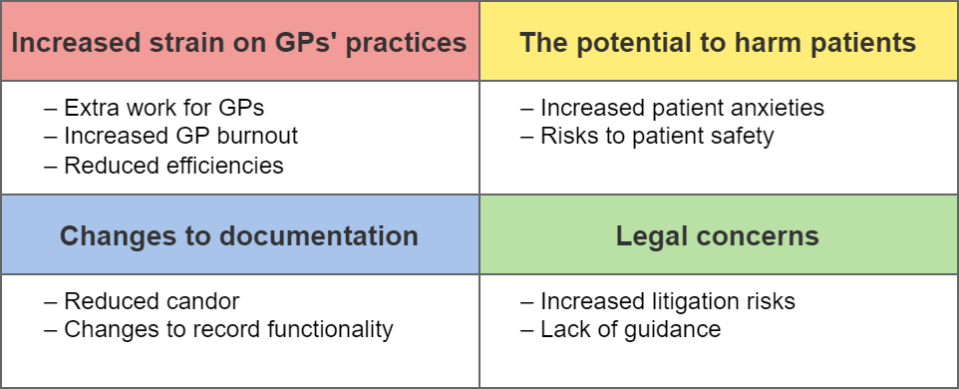
The 4 major categories of GPs’ views that were identified in relation to sharing online access to patients’ health records. GP: general practitioner.

Owing to the iterative thematic analysis process, four major categories of GPs’ views were identified in relation to sharing online access to patients’ health records: (1) increased strain on GPs’ practices, (2) the potential harms to patients, (3) changes to documentation, and (4) legal concerns (Figure 1). These categories were further subdivided into 9 themes, which are described in subsequent sections with illustrative comments; the numbers in parentheses are identifiers that ascribe comments to individual participants.

### Increased Strain on GPs’ Practices

#### Extra Work for GPs

A dominant concern was that ORA would be “extremely time consuming” and “create a lot of extra work.” Many comments were strongly worded, for example, “another disaster which will take up GP time” (#116). Participants frequently described an experienced uptick in patient contact driven by patient “confusion” and the need to “translate” clinical information in records so that patients could understand it, for example:

I have spent 60 minutes over the course of a couple [word missing] trying to explain blood results to a patient which he was following on his online records and there was a great effort involved on my part clarifying the explanations to his satisfaction.

#280

No objections to patients have access, in principle. However, it has led to increased amount of time spent with fielding phone calls about content of the records.

#360

Many hours of time dealing with patients’ misinterpretation of medical information already.

#106

Already seeing a marked increase in callback requests from patients viewing minor “abnormalities”... Concerns that this problem will be exacerbated as patients have access to their full clinical records.

#339

In contrast, a few GPs expressed different experiences with the practice:

Generally helpful and slightly decreases our work.

#132

Occasional queries so far about records and comments.

#382

Other participants *forecast* that ORA would lead to extra work, again with many comments similarly connecting this to a perceived increase in patient contact because of “confusion” or “misinterpretation,” which was often couched as “timewasting,” for example:

We have more than enough work to do without having to field scores of queries about the contents of the records.

#20

Will create unnecessary work as patients will be contacting to discuss every little detail.

#297

Some participants offered more moderate views but anticipated, or experience, “difficult patients” would drive extra work via increased contact, for example:

Lots of queries about mildly abnormal path lab reports. The sort of patients likely to access their records are also those who also tend to be rather neurotic (I’m generalising here, but this has been my experience so far).

#71

#### Reduced Efficiencies

Many comments more explicitly expressed concerns about the prospect of reduced efficiencies because of ORA, for example: “There is the potential for a massive increase in inefficient workload with this measure” (#76). Concerns about experienced or anticipated “unnecessary work” were described as contributing to practice inefficiencies, for example:

We are short-staffed already and unable to recruit to fill vacancies...Having to spend time responding to patient queries about medical records has the potential to be a major destabilizing factor in our practice, as these will not be queries that admin staff can deal with. It is likely to result in a longer wait for appointments as more clinical time is spent on this.

#350

Some comments expressed explicit or implied doubts about whether the NHS had “extra resources” to cope with comments robustly expressed, for example:

Primary care is knackered, help!!

#29

It’s going to be a nightmare! Sort the basics first - IT, staffing etc. We don’t have the resources to deal with the consequences of this yet.

#262

I am not sure whether current staffing levels and IT systems support full online access.

#222

#### Increased GP Burnout

Many comments suggested patient access might exacerbate burnout, for example:

Too late now but we don’t have the time or energy for this!!...I’m really worried about the extra workload it will create - will tip primary care over the edge I’m afraid!!!

#29

Oh good more queries about unimportant stuff to keep me busy when I'm already working with inadequate clinical & admin cover due to COVID and vacancies.

#249

We need this like a hole in the head...This nonsense in our wealthy practice is threatening to overwhelm us.

#300

However, not all GPs agreed, although fewer were more optimistic, for example:

Feels a bit threatening but probably helpful in long run.

#248

We should not be afraid.

#156

Happy for patients to see their records.

#225

### The Potential to Harm Patients

#### Increased Patient Anxieties

Another dominant theme was the potential of ORA to harm patients, in particular, to cause “upset,” “anxieties,” and “emotional harm.” Concerns were often described as emerging because of perceived or experienced patient confusion, for example:

They read things they don’t understand so get worried.

#77

For [a] majority I feel it will cause more worry, confusion and damage the relationship between doctor and patient.

#283

Many GPs identified “borderline test results” as a particular source of anxiety, and some comments linked this to an increased workload, for example:

Most people are anxious regarding their health conditions and looking record will make them more anxious especially borderline pathology results.

#286

Borderline results which are normal will provoke enquiries and the big risk is massive workload to reassure patients that borderline results in fit healthy individuals don’t need investigating.

#147

#### Risks to Patient Safety

Many GPs expressed “concern over safeguarding” (a term used commonly in the United Kingdom and Ireland referring to patient safety, which relates specifically to abuse and neglect, and the ability to live safely), although comments were frequently brief or truncated, for example:

I worry about a significant minority where this will cause harm, especially those with mental health conditions and safeguarding issues.

#390

However, some participants identified domestic abuse or coercive relationships as a source of risk, for example:

Once had a problem where a partner accessed a patient’s record which had domestic violence as a problem as she had disclosed this...don’t think it made her care any safer.

#243

What if someone is forced to give access to another person?

#120

Increases safeguarding risks - poorly thought out/not considered. e.g. If a patient found out the details of their ex-wife's new partner (phone number, name etc) or their ex-partner’s new phone number this would increase risk of domestic abuse/stalking.

#326

In contrast, 2 participants suggested that access might offer, at least the theoretical potential, the promotion of safety through patient identification of mistakes in documentation:

They can spot errors in records. They’ll be more informed about their health.

#338

If we had lots of time patient access would be a good way to share information and to correct occasional important errors in the record but we aren’t funded or staffed to have the time required for this to work well.

#268

### Changes to Documentation

#### Reduced Candor

A dominant view was that GPs would need to “limit” what they wrote or “not be completely honest” with the knowledge that patients could access their free-text entries. The need to omit clinical judgments or differential diagnoses to avoid patient distress was a particular concern, for example:

Clinicians less likely to add personal comments and opinion from their gut instinct.

#162

I think it will generate more work and anxiety and impede full record keeping.

#261

[T]end to add a differential diagnosis and potential further investigations which aid other doctors dealing with the patient in my absence. However, these may fuel anxiety if misunderstood by patients. I would therefore need to deliberately decrease the quality of my consultation notes to make them more patient-friendly.

#398

Some comments reflected changes relating to “careful wording” with respect to sensitive information, to reduce anxieties, or to avoid information that patients might find offensive. Comments frequently reflected the need to compromise the content of documentation, for example:

Makes it much harder for clinicians to make meaningful comments about their observations if these may be viewed in a negative light by patients.

#34

We need to be mindful what patients can access. As a result, I limit what I write in my consultations and limit or avoid my thoughts (I write only facts and observations) which would normally be helpful for the next doctor treating the same ailment (i.e., if patient malingering, suspicion of safeguarding, advanced disease progression).

#85

#### Changes to Record Functionality

Multiple comments expressed more general concerns about changes in the functionality of GP records because of patient access, for example:

I write things in there for my benefit and other doctors’ benefits not for patients to read.

#343

Some GPs described the need to use records for their own “musings” or to support their thought processes, for example:

I also feel there should be the ability for GPs to make comments or aide-memoires that are not visible to patients.

#314

Free text is also a way that GPs communicate with each other and is especially useful when multiple doctors take care of one patient. It would be a pity to lose that. There should be the option of typing text which patients cannot see.

#359

### Legal Concerns

#### Increased Litigation Risks

Another theme was related to “threats of legal action.” Some GPs conveyed the concern that ORA would encourage “litigious” patients and lawyers “fishing for errors,” for example:

No win no fee lawyers would be interested to find any mistakes in care as in insurance claims.

#15

I worry that patients will send copies to medico legal companies to trawl through looking for mistakes.

#56

So far, I have had three formal complaints generated by full record access. One has taken up litigation and it was clearly used as a “fishing” exercise to see if a case could be brought.

#64

We live in a parasitical legal system where people play the fruit machine with contingency fee-based lawyers.

#300

One GP worried about legal consequences resulting from patients’ desire to change their documentation:

This may lead to patients requesting omission of certain facts which will not be an option as medical notes should not be tampered with.

#238

#### Lack of Guidance

Some comments described a lack of legal guidance associated with managing records that patients could now read or to which they could contribute, for example:

They are already leaving comments in the record, and we have no idea how to handle this – it is rarely helpful, creates more admin work, and potentially they can leave comments which will not be read by the clinician leading to medico legal problems.

#103

Drawing on his own concerns about an estranged father requesting access to his 15-year-old’s online records, 1 GP expressed concerns about the lack of clear guidance for GPs:

He is taking legal action against us for refusing him access...We have sought guidance of LMC [Local Medical Committee], NHS England and MDU [Medical Defense Union] etc. - no clear precedent as these issues were “not anticipated”. The legal implication rests directly with us personally as clinicians - that is wholly unfair - we have had no training and there is no guidance in this area.

#72

## Discussion

### Principal Findings

This qualitative study provides new insights into how GPs in England view the impact of patient access to their full electronic health records, including free-text entries. Comments were classified into four major themes: (1) increased strain on GPs’ practices, (2) the potential to harm patients, (3) changes to documentation, and (4) legal concerns. GPs believed that access would lead to extra work for GPs, reduced efficiency, and increased burnout. Participants also believed that access would increase patient anxiety and incur safeguarding risks. As a consequence of the ORA, GPs reported that they either had or would change the way they wrote in the record, reducing candor, and changing the functionality of the record. Anticipated legal concerns encompassed fears about increased litigation risks and lack of guidance to GPs about how to manage documentation that would be read by patients and potential third parties.

This qualitative study supports previous research showing that GPs in England are skeptical that the benefits of ORA outweigh perceived concerns. In addition, the findings are strikingly similar to those of studies conducted among clinicians in other countries [23,24,26-29,37,47]. A dominant theme was the concern that ORA would lead to increased strain on the already strained practices of GPs. Highlighted sources of strain included additional workload for GPs addressing potential patient confusion about note content, failure to make the best use of GP time or other NHS resources, increased risk of GP burnout, and exacerbation of existing difficulties with staff retention and morale. Frustrations around the capacity to manage perceived increases in workload and navigate necessary changes to workflow have been reported in other recent research undertaken with English, clinical, and nonclinical staff before the NHS England announcement [6,37]. Nonetheless, it is important to note that this study was administered during the COVID-19 pandemic, during a period in which general practice was operating under unprecedented conditions, and also in the wake of the NHS England announcement, which has been subject to considerable debate and delay.

Our respondents’ concerns could be overestimated. Studies conducted in countries with more established ORA systems indicate that over time, levels of strain in GP’s practices appear to be much lower than originally feared. NHS Digital reported that between 2021 and 2022, GP surgeries taking part as “early adopters” in the accelerating patient records access program in England initially raised concerns regarding the potential for the program to increase the volume of patient enquiries [48]. Despite this, staff from these early adopter sites subsequently did not report an increase in workload, and some reported perceived reductions in workload because they no longer had to deal with requests to grant access to records [48].

However, concerns about increased work relating to enquiries from patients viewing test results via ORA might be warranted. A recent study investigating the association of immediate electronic release of test results and implications on clinical workflow in the United States reported that messages sent by patients within 6 hours of reviewing a test almost doubled (from a median of 77.5, IQR 13.75-105.25, to 146, IQR 12.0-169.0) when they were released to the patient portal before clinician review [49]. Other studies in the United States have shown that ORA was associated with increased use of clinical services compared with patients who did not have online access [50]. It is worth adding that differences in the design of patient portals and ORA, for example, regarding how much information patients have access to, when they can access it, and safeguarding measures in place, may have an impact on clinicians’ opinions on ORA or on how patients use follow-up clinical services. Few studies have explored this so far, but this work indicates both portal design and what patients are told about access, likely affecting patient adoption and clinician acceptance [51-53].

GPs also believed that patients would experience increased harm from accessing their online records, a concern shared by patients in a qualitative study in England [5]. Other studies in Nordic countries have also shown that health care professionals have similarly strong concerns [28-31]. Common concerns are that patients would become anxious when reading results before speaking to a clinician or misunderstanding the content of the record [28]. However, ORA might also function as a safety mechanism. In an analysis of 20 randomized clinical trials related to sharing clinical notes, involving 17,387 patients, Neves et al [20] concluded that sharing electronic health records could improve patient safety. Few GPs in this study mentioned the potential benefits of patients identifying errors, and more awareness of the potential for this patient feedback loop on care and safety through ORA is needed. It is common for patients to find errors in their records; in a US study, 21.1% (N=4830) of note readers found a mistake in their notes [54], and patients and their families have the potential to contribute to improved patient safety when given access to full records [55].

GPs expressed concerns that ORA would lead to documentation changes, including reduced candor in free-text entries, and to significant changes in the function of the records. Anticipating patient anxiety and confusion, GPs feared the “dumbing down” of documentation, and some GPs reported being less candid with the knowledge patients might read their records. These concerns were also reported by Turner et al [6] and are similarly shared by clinicians in other countries where ORA is advanced. Studies in the United States also show that clinicians report changing how they write free-text entries after experiencing patient access [56]. For example, in a survey of primary care physicians (n=116), Ralston et al [47] found that 67% of respondents anticipated being less candid in their documentation before the implementation of open notes, dropping to 49% in 12 months after patient access.

GPs also expressed broad concerns that patient access will increase litigation risks and described a lack of guidance on how to manage emergent legal issues. Currently, we are aware of no medical malpractice cases that have emerged because of patient access to their records. Blease et al [57] argued that there is no substantial published evidence to make a decision. If GPs make changes that diminish the medical quality of documentation later, leading to errors, or if they fail to correct significant documentation errors identified by patients that later cause errors, this could exacerbate the risks of suits [57]. This concern is also closely related to patient safety; if clinicians do not feel able to write full reports in their entries, they may start communicating with colleagues through other channels, which could lead to diminished safety. For example, a GP in the study by Turner et al [6] described an instance where the respondent phoned or chatted with colleagues, rather than documenting their concerns about a patient.

### Strengths and Limitations

This study is the largest conducted to date exploring the views of GPs in England on patient access to their web-based health records, including their free-text entries. These themes support and also extend the results of earlier, smaller published surveys in England on the topic [58]. Although previous qualitative studies used focus groups or interviews exploring the views of primary care staff, including GPs [6], this survey focused exclusively on GPs currently registered and working in England. The use of a web-based survey may have facilitated more candid feedback, as reflected in the strength of our participants’ responses.

However, the survey had limitations related to the use of a nonprobability sample via Doctors.net.uk. Although we strove to stratify the sample as far as possible according to geographic location, sex, and age, our respondents were restricted to those GPs who used Doctors.net.uk, including during the administration of the survey. Comments were often brief owing to restrictions on web-based design. In addition, owing to the survey design, and because (at the time of survey distribution) GPs could offer patients online access on a patient-by-patient basis (providing access only when patients requested it), it was unclear from many comments whether participants’ responses were grounded in experience (even limited experience) or in speculation. In addition, response biases may have influenced the findings, and it is also possible that those responses were GPs with strong views rather than individuals who were more disinterested in the topic. For example, it is possible that GPs who are more worried about imminent patient access are more likely to respond.

We recommend that ongoing qualitative research probe the views of GPs after ORA has been implemented and that they have experience in the practice. We plan a follow-up web-based mixed methods survey among those who participated in the survey and who have agreed to be contacted at a later date. More robust, nationally representative surveys are required to obtain a clearer picture of GPs’ views and experiences. There is a need for further verification of what could arguably be viewed as anecdotal evidence and for more robust experimental studies examining workload impacts using objective measures. Finally, we urge further extensive survey research to explore the attitudes and opinions of patients in England after they have experience with accessing their web-based records.

### Conclusions

This descriptive analysis provides exploratory insights into the views of 224 GPs in England regarding the impact of ORA on patient care and their practice. GPs identified many concerns related to the potential for access to increase workload, harm patients, compromise the quality of records, and increase legal risk. As we consider these findings, we cannot help but reflect on the commonality of clinician concerns across national boundaries [23,24,27,58].

As with all innovations, the ORA invites new challenges [59,60]. With the evolving functionality of web-based health records [61], GPs in England, similar to their counterparts in other countries, will need advice and guidance, including formal training, to become more comfortable writing and talking about documentation that patients will now read, including how to manage patient concerns and feedback [62-65].

Further qualitative research is required to understand the perspectives of patients in England regarding their experiences while accessing their web-based records. Beyond the growing body of cross-cultural survey findings with ORA, there is a greater need to explore objective measures of the impact of patient access to their records on health outcomes, clinician workload, and changes to documentation.

## Appendix

Multimedia Appendix 1 Medical records access survey for general practitioners.

Multimedia Appendix 2 Comparison of general practitioners who did and did not provide comments to each open comment question.

Multimedia Appendix 3 Raw data.

## Abbreviations

GMC: General Medical Council
GP: general practitioner
NHS: National Health Service
ORA: online record access

## Data Availability

Raw data are available in Multimedia Appendix 3.
