# Supplementary material for: Patient Online Record Access in English Primary Care: Qualitative Survey Study of General Practitioners’ Views
Source: J Med Internet Res. 2023 Feb 22;25:e43496. doi: 10.2196/43496 (PMC9996425; doi:10.2196/43496)
Supplement: Multimedia Appendix 2 [file jmir_v25i1e43496_app2.docx]

**Appendix 2: Comparison of GPs who did and did not supply comments to each open comment question.**

**Table A: Comparison of GPs who did and did not supply qualitative comments to Q1 “Please add any additional comments you might have about the information your patients can access.”**

| **Characteristic** | **Qualitative**  **Comments (N=154)**  n (%) | **No qualitative**  **Comments (N=246)**  n (%) | **Comparison (Chi-square)** |
| --- | --- | --- | --- |
| Female | 72 (48.32%) | 89 (37.24%) | χ2 (1, *N* = 388) = 4.64  *p* = .031 |
| Hours worked> 40 hours per week | 40 (25.97%) | 65 (26.42%) | χ2 (1, *N* = 400) = .01  *p* = .921 |
| Age > 40 | *130* (84.42%) | *209* (84.96%) | χ2 (1, *N* = 400) = .02 *p* = .883 |
|  |  |  |  |
| GPs offering full ORA with > 51-100% of patients | *44 (34.11%)* | *67 (35.64%)* | χ2 (1, N = 317) = .08  p = .779 |

**Table B: Comparison of GPs who did and did not supply qualitative comments to Q2 “Do you have any additional comments about the impact to patients accessing their full GP health record online?”**

| **Characteristic** | **Qualitative**  **Comments (N=78)**  n (%) | **No qualitative**  **Comments (N=322)**  n (%) | **Comparison (Chi-square)** |
| --- | --- | --- | --- |
| Female | 35 (46.05%) | 126 (40.38%) | χ2 (1, *N* = 388) =.81  *p* = .369 |
| Hours worked> 40 hours per week | 20 (25.64%) | 85 (26.40%) | χ2 (1, *N* = 400) = .02  *p*=.892 |
| Age > 40 | *70* (89.74%) | *269* (83.54%) | χ2 (1, *N* = 400) =1.87  *p*=.17 |
|  |  |  |  |
| GPs offering full ORA with > 51-100% of patients | *24 (33.80%)* | *87 (35.37%)* | χ2 (1, N = 317) = .06  p=.808 |

**Table C: Comparison of GPs who did and did not supply qualitative comments to Q3 “Do you have any additional comments about the impact of patient access to their full online health records on your practice?”**

| **Characteristic** | **Qualitative**  **Comments (N=159)**  n (%) | **No qualitative**  **Comments (N=241)**  n (%) | **Comparison (Chi-square)** |
| --- | --- | --- | --- |
| Female | 60 (38.46%) | 101 (43.53%) | χ2 (1, *N* = 388) =.99  *p* = .32 |
| Hours worked> 40 hours per week | 44 (27.67%) | 61 (25.31%) | χ2 (1, *N* = 400) = .28  *p*=.599 |
| Age > 40 | *141* (88.68%) | *198* (82.16%) | χ2 (1, *N* = 400) =3.15  *p*=.08 |
|  |  |  |  |
| GPs offering full ORA with > 51-100% of patients | *45 (36%)* | *66 (34.38%)* | χ2 (1, N = 317) = .09  p=.767 |

**Table D: Comparison of GPs who did and did not supply qualitative comments to Q4 “Do you have any additional comments, anecdotes, and/or expectations to share about patients' online access to their full online health record including clinicians' free text entries?”**

| **Characteristic** | **Qualitative**  **Comments (N=62)**  n (%) | **No qualitative**  **Comments (N=338)**  n (%) | **Comparison (Chi-square)** |
| --- | --- | --- | --- |
| Female | 31 (50.82%) | 130 (39.76%) | χ2 (1, *N* = 388) =2.59  *p* = .11 |
| Hours worked> 40 hours per week | 17 (27.42%) | 88 (26.04%) | χ2 (1, *N* = 400) = .05  *p*=.82 |
| Age > 40 | *55* (88.71%) | *284* (84.02%) | χ2 (1, *N* = 400) =.89  *p*=.345 |
|  |  |  |  |
| GPs offering full ORA with > 51-100% of patients | *17 (34%)* | *94 (35.21%)* | χ2 (1, N = 317) = .03  p=.869 |
